# Separation of breast cancer and organ microenvironment transcriptomes in metastases

**DOI:** 10.1186/s13058-019-1123-2

**Published:** 2019-03-06

**Authors:** Mohammad A. Alzubi, Tia H. Turner, Amy L. Olex, Sahib S. Sohal, Nicholas P. Tobin, Susana G. Recio, Jonas Bergh, Thomas Hatschek, Joel S. Parker, Carol A. Sartorius, Charles M. Perou, Mikhail G. Dozmorov, J. Chuck Harrell

**Affiliations:** 1 Department of Pathology, Virginia Commonwealth University, Richmond, VA USA; 2 Integrative Life Sciences Program, Virginia Commonwealth University, Richmond, VA USA; 3 C. Kenneth and Dianne Wright Center for Clinical and Translational Research, Virginia Commonwealth University, Richmond, VA USA; 4 Department of Oncology and Pathology, Cancer Center Karolinska, Karolinska Institutet and University Hospital, Stockholm, Sweden; 5 Department of Genetics, Lineberger Comprehensive Cancer Center, University of North Carolina at Chapel Hill, Chapel Hill, NC USA; 6 Department of Pathology, University of Colorado Anschutz Medical Campus, Aurora, CO USA; 7 Department of Biostatistics, Virginia Commonwealth University, Richmond, VA USA; 8 Massey Cancer Center, Virginia Commonwealth University, Richmond, VA USA; 9 Department of Pathology and Laboratory Medicine, Lineberger Comprehensive Cancer Center, University of North Carolina at Chapel Hill, Chapel Hill, USA

**Keywords:** Breast cancer, Luciferase, Metastasis, Microenvironment, Patient-derived xenograft, RNA sequencing

## Abstract

**Background:**

The seed and soil hypothesis was proposed over a century ago to describe why cancer cells (seeds) grow in certain organs (soil). Since then, the genetic properties that define the cancer cells have been heavily investigated; however, genomic mediators within the organ microenvironment that mediate successful metastatic growth are less understood. These studies sought to identify cancer- and organ-specific genomic programs that mediate metastasis.

**Methods:**

In these studies, a set of 14 human breast cancer patient-derived xenograft (PDX) metastasis models was developed and then tested for metastatic tropism with two approaches: spontaneous metastases from mammary tumors and intravenous injection of PDX cells. The transcriptomes of the cancer cells when growing as tumors or metastases were separated from the transcriptomes of the microenvironment via species-specific separation of the genomes. Drug treatment of PDX spheroids was performed to determine if genes activated in metastases may identify targetable mediators of viability.

**Results:**

The experimental approaches that generated metastases in PDX models were identified. RNA sequencing of 134 tumors, metastases, and normal non-metastatic organs identified cancer- and organ-specific genomic properties that mediated metastasis. A common genomic response of the liver microenvironment was found to occur in reaction to the invading PDX cells. Genes within the cancer cells were found to be either transiently regulated by the microenvironment or permanently altered due to clonal selection of metastatic sublines. Gene Set Enrichment Analyses identified more than 400 gene signatures that were commonly activated in metastases across basal-like PDXs. A Src signaling signature was found to be extensively upregulated in metastases, and Src inhibitors were found to be cytotoxic to PDX spheroids.

**Conclusions:**

These studies identified that during the growth of breast cancer metastases, there were genomic changes that occurred within both the cancer cells and the organ microenvironment. We hypothesize that pathways upregulated in metastases are mediators of viability and that simultaneously targeting changes within different cancer cell pathways and/or different tissue compartments may be needed for inhibition of disease progression.

**Electronic supplementary material:**

The online version of this article (10.1186/s13058-019-1123-2) contains supplementary material, which is available to authorized users.

## Background

It is well accepted that there are many different breast cancer subtypes, which have a wide range of prognoses [1–5]. Nearly all patients that die from breast cancer succumb to the disease due to the expansive growth of metastatic cells within vital organs. The spread of cancer cells away from the primary tumor depends on their ability to intravasate into the vasculature, survive in the circulation, extravasate into a foreign tissue microenvironment, and proliferate into a secondary mass. These properties are variable across different breast cancer subtypes, which give rise to different metastatic organ distributions, expansive latencies, and overall survival outcomes [6].

Knowledge of each phase of the metastatic cascade has dramatically increased over the past 20 years. Breast tumors do not need to form an overt mass prior to dissemination, and often, metastases have already spread to vital organs at the time of initial primary tumor diagnosis [7]. Triple-negative breast cancer (TNBC) cells can perform vascular mimicry, interdigitate within endothelial cells, and spread very early in the development of a malignant lesion [8–10]. Once in the lymphatic or blood vascular circulation, the cells can travel as single cells or emboli which may or may not result in clonal or polyclonal secondary metastatic tumors. Successful metastatic extravasation into vital organs is a rate-limiting step for many cancer cells [11] and does occur in each subtype of breast cancer. The biological mechanisms that promote successful metastatic expansion are found within the cancer cells and facilitative organ microenvironment. A better understanding of how different metastatic microenvironments contribute to the expansion of micro-metastases into macro-metastases will allow more specific targeting of the microenvironment to prevent the expansion of metastatic disease.

To elucidate cell-specific gene expression in metastases, previous studies have performed laser capture microdissection of frozen tissue sections, or antibody-based cell sorting of live cells, followed by gene expression analyses [12, 13]. Alternatively, human and mouse-specific gene expression microarrays have been used on xenografts derived from cancer cell lines [14, 15]. Bioinformatic analyses of sorted tumor samples, or bulk tissues from patients or transgenic mouse models, have identified that the majority of cancer-driving mutations and DNA copy number changes are present in the primary tumor and largely maintained throughout disease progression [5]. It is now important to develop RNA sequencing datasets and analysis pipelines to more accurately define both cancer and microenvironmental contributors to metastatic colonization. In these studies, we focus on characterizing the metastatic properties of 14 distinct patient-derived xenografts (PDX) that represent estrogen receptor (ER) positive, TNBC, and human epidermal growth factor receptor 2 (HER2)-positive disease. Given the lack of treatment options for TNBC, we primarily focus on understanding the mechanisms used by these cells to grow in the liver and lung. Interestingly, a set of common genetic signatures was identified as being upregulated in multiple basal-like metastases, targeting the most upregulated pathway with FDA-approved drugs reduced cancer cell viability when tested on PDX spheroids. In addition, an inflammatory response genomic program was upregulated by the liver microenvironment in response to the invading cancer cells. We hypothesize that this information can be used to help develop metastasis-targeted therapeutics to inhibit the growth of disseminated cells.

## Methods

### Development of PDX metastasis models

PDX models were obtained through material transfer agreements with Washington University in Saint Louis (WHIM2, WHIM30), the University of Utah/Huntsman Cancer Institute (HCI01, 02, 03, 04, 08, 09, 10, 11, 13, 16), and the University of Colorado (UCD18, UCD52). After successful growth of the PDX cells or tumor fragments in the fourth mammary fat pad of non-obese diabetic severe combined immunodeficiency (NOD-SCID-gamma/NSG) mice, tumors were collected and digested into a single-cell suspension as described previously [16, 17] and then incubated with lentivirus encoding for green fluorescent protein and luciferase (GFP+Luc) (BLIV101PA-1; Systems Biosciences). After selection of the transduced cells with flow cytometry, the cells were serially passaged in the mammary gland. All experiments utilized 500,000 cancer cells to initiate mammary tumors or metastases. Throughout the in vivo studies that investigate rate and location of metastases, mice were routinely imaged using an IVIS Spectrum In Vivo Imaging System (IVIS200), after injection of d-luciferin (15 mg/ml) (Gold Biotechnology). Metastases were quantified with ex vivo imaging of each organ at necropsy. Sites of relapse of the PDX tumors were compared with previous publications or from personal communications with Dr. Alana Welm.

### Immunohistochemistry and immunofluorescence

To determine standard clinical biomarker status for the PDXs, mammary tumors were fixed in 10% formalin overnight, paraffin-embedded, and cut into 6-μm sections. Slides were subjected to heat-induced epitope retrieval with a pressure cooker in pH 9 Tris-EDTA buffer. Antibodies used were from the following: Abcam: estrogen receptor (ER) (SP1 ab16660); Biolegend: Alexa Fluor 488 Cytokeratin (628608) and Alexa Fluor 594 Vimentin (677804); Cell Signaling Technology: HER2 (2242), Ki67 (D2H10), PR (8757), S100A9 (73425), and Vimentin (D21H3); ThermoFisher: Cytokeratin (PA1-27114); Millipore: LCN2 (AB2267); and Novus: SMA (NB600-531). Antibodies used for immunohistochemistry were incubated overnight at 4 °C and combined with the DAKO EnVision rabbit secondary antibody kit. All images were collected with a Zeiss AxioLab upright microscope and Zeiss AxioCam ICc 5, and Zen2 software.

#### RNA sequencing and availability of data

Tissues were flash frozen, and RNA was prepared with the Qiagen RNeasy mini kit. The KAPA Stranded mRNA-Seq kit was used for library preparation, and samples were sequenced on the Illumina Hi-Seq 2500 according to Illumina sequencing-by-synthesis protocol. One hundred twenty-five-base pair paired-end reads were generated, yielding on average 42 M reads per sample. The dataset supporting the conclusions of this article are available in the NCBI Gene Expression Omnibus (GEO Accession GSE118942). All library preparations and sequencing were performed by the Brigham Young University DNA Sequencing Core. Reads were aligned to a custom concatenated reference genome consisting of human, mouse, and viral genomes. The GRCh38 human genome that includes viral sequences (GRCh38.d1.vd1.fa.tar.gz) was retrieved from NCI’s Genomic Data Commons at https://gdc.cancer.gov on January 3, 2017. The corresponding annotation file (gencode.v22.annotation.gtf.gz) was downloaded from the same site on 1/4/17. The GRCm38 M12 Gencode release, primary assembly mouse genome was retrieved from http://www.gencodegenes.org on January 3, 2017 along with the corresponding primary assembly annotation file from the same site on January 4, 2017. Finally, the genome of Xenotropic murine leukemia virus (XMLV) (accession #JF274252.1) was downloaded from NCBI’s Nucleotide database, https://www.ncbi.nlm.nih.gov on February 28, 2017. Chromosomes were labeled with organism-specific prefixes before concatenation in a similar fashion to the method described by Callari et al. [18], which recently reported custom concatenated reference genomes to outperform other methods in classifying RNA from different species in PDX models.

#### RNA-Seq pre-processing and quality control

FastQC v.0.11.5 [19] was used for quality control. STAR v2.5.2b [20] was used to index the concatenated reference genome prior to alignment. The FASTQ files were aligned to the concatenated genome using STAR with the following parameter settings: --outSAMtype BAM Unsorted --outSAMorder Paired --outReadsUnmapped Fastx --quantMode TranscriptomeSAM --outFilterMultimapNmax 1. The number of unmapped reads and reads mapped to human, mouse, and viral genomes were obtained through an in-house bash script utilizing SAMTools v1.3.1 [21]. Human and mouse percentages were calculated using the total number of reads aligned to human or mouse only, and do not include unmapped reads or reads aligned to viral genomes. Read counting to obtain gene expression values from the aligned BAM files utilized a transcript reference file created from the concatenated genome and was performed using the Salmon v0.8.2 “quant” algorithm [22] with the library type set to “IU.” The counts were imported into R v.3.4.0 using the tximport [23] R package v.1.4.0. Transcripts per million (TPM) expression values were calculated using human and mouse transcript counts separately. This effectively normalizes the gene expression by the varying percent of human and mouse genomic content. Log2-transformed TPM values were used for all analyses except the PAM50 subtyping, which used unlogged TPM values. Genes were filtered to remove those with a Log2 TPM of zero across all samples. Quality control steps were taken to ensure data integrity before and after alignment. Evaluating the number of reads sequenced in each FASTQ file revealed several samples that had fewer than 15 million sequenced reads. These files were re-sequenced, and resulting data from the two runs were merged as BAM files after alignment using SAMTools v1.3.1 merge function (Additional file 1) indicates which files were run a second time. The merged BAM files were then utilized in the rest of the pipeline for these samples. A second quality control step utilized Pearson’s correlation of the Log2 TPM expression profiles for all sample pairs. This revealed two samples that clustered unexpectedly with different cell lines where the expression profiles were clearly different from the labeled cell type. Upon further investigation, it was found these samples were right next to each other in the tray and the labels were accidentally swapped. For all analyses, these sample labels are corrected to refer to the correct cell type.

#### PAM50 subtype classification

Prior to subtyping, the PDX samples were filtered to those with more than 10 million mapped human reads, which resulted in 101 samples for subtyping. PAM50 subtypes were identified for each sample from the TPM values using the “molecular.subtyping” function of the genefu v2.11.2 package [24] in R with the “sbt.model” set to “pam50”.

#### TCGA/PDX integration

Gene expression for TCGA-BRCA samples was obtained via the TCGABioLinks v2.5.9 Bioconductor R package [25] using the GDCquery function with the following settings: project = c(“TCGA-BRCA”), file.type = “rsem.genes.results”, platform = “Illumina HiSeq”, legacy = TRUE, sample.type=“Primary solid Tumor”, data.category = “Gene expression”, data.type = “Gene expression quantification”, experimental.strategy = “RNA-Seq”. The RSEM scaled estimates were converted to Log2 TPM values as described in Li et al. [26]. PAM50 subtypes for each TCGA patient were obtained from Ciriello et al. [3] resulting in a list of 817 patients from the TCGA data with a PAM50 subtype. Filtering the PDX samples to remove all normal mouse samples and all samples with less than 10 million human reads left 101 PDX samples for integration with TCGA data. The Log2 TPM values of the TCGA and PDX cohorts were merged, and the data matrix was filtered to contain expression for the PAM50 genes. Gene expression values were then upper quantile normalized and row-median centered prior to clustering. Merged TCGA/PDX data were clustered using the ComplexHeatmap v1.14.0 package [27] in R with Pearson’s correlation as the distance metric and ward.D2 as the linkage method.

#### Differential expression analysis

All differential gene expression analyses were done using the DESeq2 v1.16.1 R package with raw read counts as input. Prior to DESeq2 analysis, genes with zero expression across all samples were removed, and only genes having average expression larger than 0.5 in at least 10% of the samples and with the ratio of maximal/minimal intensity larger than 0.5 were kept. This resulted in a lower number of genes for analysis by DESeq2, which reduced multiple testing correction burden and analysis time. The exact number of genes considered by DESeq2 after gene filtering varied depending on the samples included in the contrast. Gene expressions were adjusted by percent of human or mouse aligned reads by including the appropriate percentage in the DESeq2 dataset design formula as “~ percent+condition”, where “percent” was the human or mouse percent of mapped reads (mouse percent was used when calculating mouse expression and human percent when calculating human expression), and “condition” is the sample condition (e.g., MGT, Met, Normal). A Benjamini-Hochberg adjusted *p* value of 0.05 or less was used as the threshold for determining a significant differentially expressed gene. For all tables, the “NA” values in the padj column indicate DESeq2 filtered these genes out of the multiple testing correction due to a low-base mean value and/or outlier values. baseMean is the normalized average read count across all samples in contrast. log2FoldChange is the Log2 of the calculated fold change.

#### Principal component analysis

PCA was run using the prcomp function on the centered and scaled, upper quantile normalized Log2 TPM expression profiles for the 2000 most variable genes for a subset of PDX samples. This subset includes one representative sample from each cell type chosen based on the highest human transcript abundance. The PCA plot was generated using ggplot2 v3.0.0 and ggrepel v0.8.0 R packages [28, 29].

#### Mouse RNA-seq expression clustering

The variance in the upper quantile normalized, Log2 TPM mouse gene expression across 42 PDX samples with greater than 50% mouse mapped reads was calculated using R’s “var” function. Hierarchical clustering of the row-median centered top 2000 most variable genes using Pearson’s correlation as the distance metric and ward.D2 as the linkage method was performed by R’s ComplexHeatmap package.

### *ANOVA* of gene expression data

TPM values from the human or mouse RNA-seq data presented herein, normalized mRNA counts from supplemental Table 7 from Siegel et al. [30], or gene expression data from Tobin et al. [31] were subjected to analysis of variance in R.

### Gene Set Enrichment Analyses

Single-sample Gene Set Enrichment Analysis (ssGSEA) was performed using the GSVA R package v.1.30.0 [32] separately for human and mouse gene expression datasets. Briefly, samples with > 50% of human (or mouse) reads were selected. The Log2-transformed TPM values were used to rank transcripts for ssGSEA analysis. The MSigDB v.5.2 [33] [34] data (> 18,000 gene signatures, https://github.com/stephenturner/msigdf) was used. Enrichment scores were clustered using Cluster 3.0 [35] and visualized using Java TreeView v.1.1.6 [36]. Differences in enrichment scores comparing average values of gene sets in mammary tumors versus metastases were calculated; the differences in average enrichment scores between mammary tumors and metastases were then combined to rank order the gene sets that were most highly upregulated in metastases across all six basal-like PDXs.

### Treatment of PDX spheroids in suspension culture

At least three different mammary tumors from seven distinct PDXs were collected, digested into a single-cell suspension as described previously [16, 17] and then plated in M87 medium. Saracatinib, bosutinib, and dasatinib were purchased from ApexBio and used at 10 μM for 72-h cytotoxicity assays. Cell viability was quantified using luciferase-based imaging, and viability of drug-treated cells was compared to that of vehicle-treated cells. All assays were performed as at least three biological replicates in triplicate.

## Results

### PDX metastasis models

**Fig. 1:**
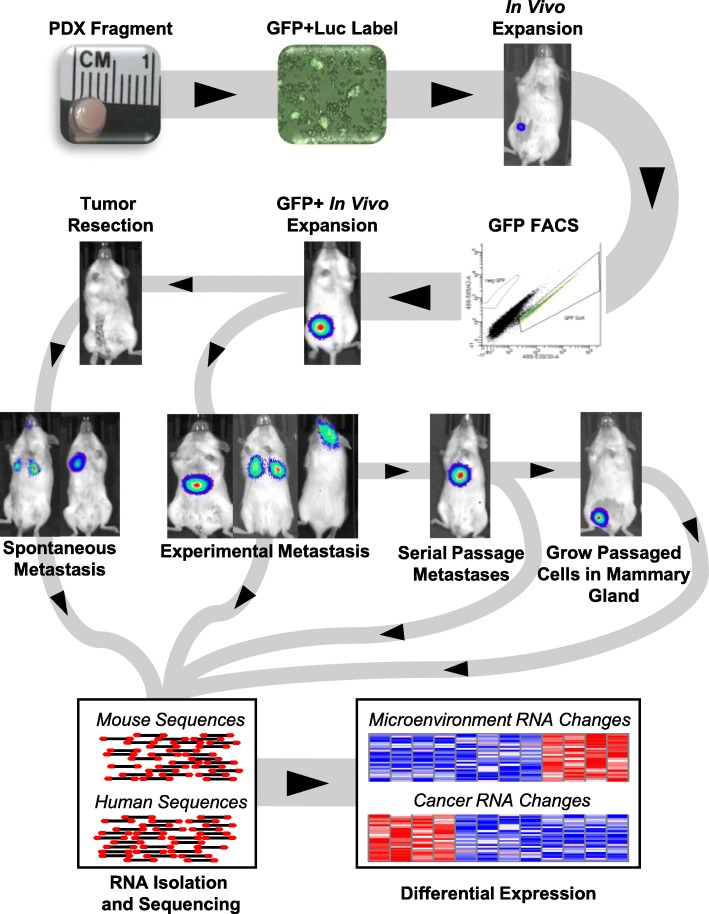
Schematic of experimental approach used to identify genomic mediators of patient-derived xenograft metastases. PDX tumors were grown in the mammary gland, extracted and digested into single-cell suspensions, and then labeled with lentivirus that encoded for green fluorescent protein and luciferase (GFP + Luc). After expansion in vivo, the cells with the brightest GFP were expanded in the orthotopic location, then used for metastasis studies. Paired-end RNA sequencing was then performed on mammary tumors, normal tissues, and brain, lung, and liver metastases. The sequencing data was then aligned to both the mouse and human reference genomes

**Fig. 2:**
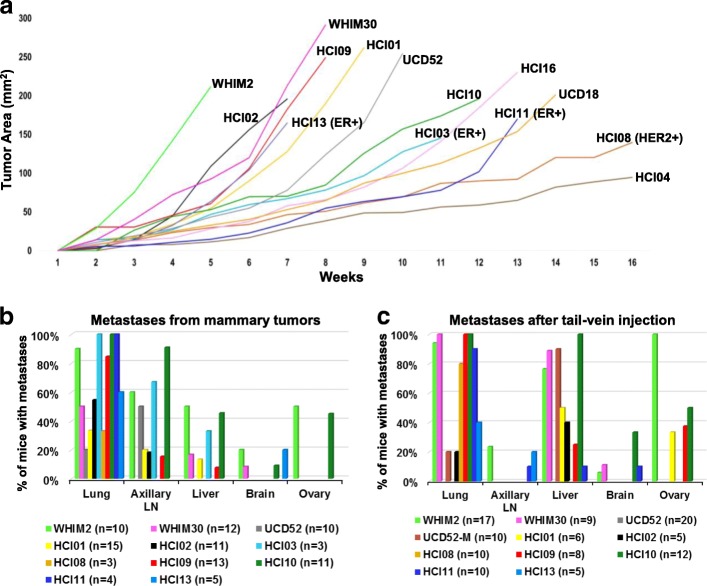
Assessment of tumor growth rates and metastatic distributions of 14 breast cancer PDXs. **a** Cancer cells were injected into the abdominal mammary gland and measured weekly for tumor growth. ER+ and HER2+ PDXs are designated, all the others are TNBC. **b** Quantification of spontaneous metastases after surgical excision of primary tumors. **c** Quantification of metastases from after tail-vein injection of PDX cells

The schematic in Fig. 1 provides an overview of the development of the PDX models and highlights how they were utilized for the studies presented herein. In total, 14 different breast cancer PDXs were transduced with lentiviral particles encoding for green fluorescent protein and luciferase (GFP+Luc): UCD18, UCD52, WHIM2, WHIM30, HCI01, HCI02, HCI03, HCI04, HCI08, HCI09, HCI10, HCI11, HCI13, and HCI16. After expansion in vivo, the GFP+ populations were collected through fluorescence-activated cell sorting, injected into the mammary glands of donor mice, and then expanded and maintained through serial passaging in the mammary gland. Immunohistochemistry for ER, PR, and HER2 was performed on mammary tumors for each PDX (Additional file 2). Three of the PDXs were ER+ and progesterone receptor positive (PR+) (HCI03, HCI11, HCI13), whereas the rest were ER−/PR−. HCI08, and, to a lesser extent, HCI04, expressed HER2 (Table 1). The mammary gland growth rate of each PDX was found to be highly variable and did not correlate with ER status (Fig. 2a).

**Table 1:**
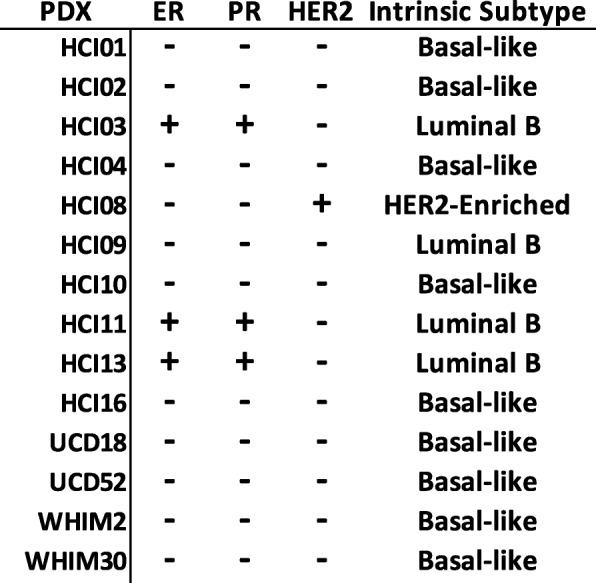
IHC characteristics and intrinsic subtype of the PDX lines

### Spontaneous metastases from mammary tumors

Several different experimental approaches were used to generate metastases that could be analyzed with RNA sequencing. First, unilateral mammary tumors from the 14 PDXs were grown and spontaneous metastases tracked with luciferase imaging. To mimic the clinical condition, survival surgeries were performed to remove primary tumors once they were 1–1.3 cm in diameter, and then mice were monitored for up to 120 days to detect metastases. At the time of surgical tumor removal, two of the PDXs had observable lymph node metastases (HCI09, HCI10). Following surgery, mice were imaged weekly for up to 16 weeks, or until moribund due to metastases. At necropsy, there was a wide variance in the organs that were colonized by the PDXs, with the exception being that all PDXs that generated metastases successfully seeded the lung and may also spread to the axillary lymph node on the same side of the mouse where the tumor was growing (Fig. 2b). Interestingly, though several PDXs effectively seeded the liver, they did not generate macro-metastases in the timeframe observed (WHIM2, HCI09). In contrast, WHIM30 and HCI10 liver metastases were large and multifocal. Brain and ovary metastases were also observed in some PDXs. Bone metastases were not routinely assessed in all models. Several models were not metastatic (UCD18 (*n* = 9), HCI04 (*n* = 4), and HCI16 (*n* = 5)).

### Metastases from systemic injections

Tail-vein injections of PDX cells were performed as a second measure of metastatic ability. As expected, the greatest overall difference between the two experimental approaches was that the axillary lymph node, which drains the abdominal mammary gland, had significantly fewer metastases in tail-vein injected mice. In contrast, liver and ovary metastases were more frequent after tail-vein injection (Fig. 2c). The same PDXs which failed to produce spontaneous metastases from primary tumors also failed to generated metastases from tail-vein injections (*n* > 3). Since spontaneous metastases from mammary tumors and tail-vein injections failed to yield consistent brain metastases, we performed intracardiac injections as this approach has been shown to yield cerebral metastases in other studies [37–39]. Successful injections of either WHIM2 or WHIM30 yielded brain metastases in 100% of the mice (Additional file 3). Comparing the patient data from which the PDX samples were derived, the PDX experiments and the organs in which metastases were found were similar; interestingly, the HCI04 PDX did not generate metastases and none were observed in the patient (Additional file 4).

### Serial passaging of metastases to heighten organ tropism

We sought to generate PDX models that were more overtly metastatic to the liver. Liver metastases were generated via liver parenchymal injections or portal vein injections with WHIM2, WHIM30, HCI10, and UCD52, and then serially passaged through multiple rounds of selection in the liver. Similar to a previous report with PDXs [40], the majority of the PDXs did not become more metastatic, except for UCD52, which resulted in liver tropic cells (UCD52-M) that homed to the liver in 90% of tail-vein injected mice, as compared to 0% of the parental PDX (Fig. 2c, Additional file 5). Spontaneous metastases from UCD52-M mammary tumors after resection were not thoroughly assessed.

### PDX metastasis RNA sequencing dataset

**Fig. 3:**
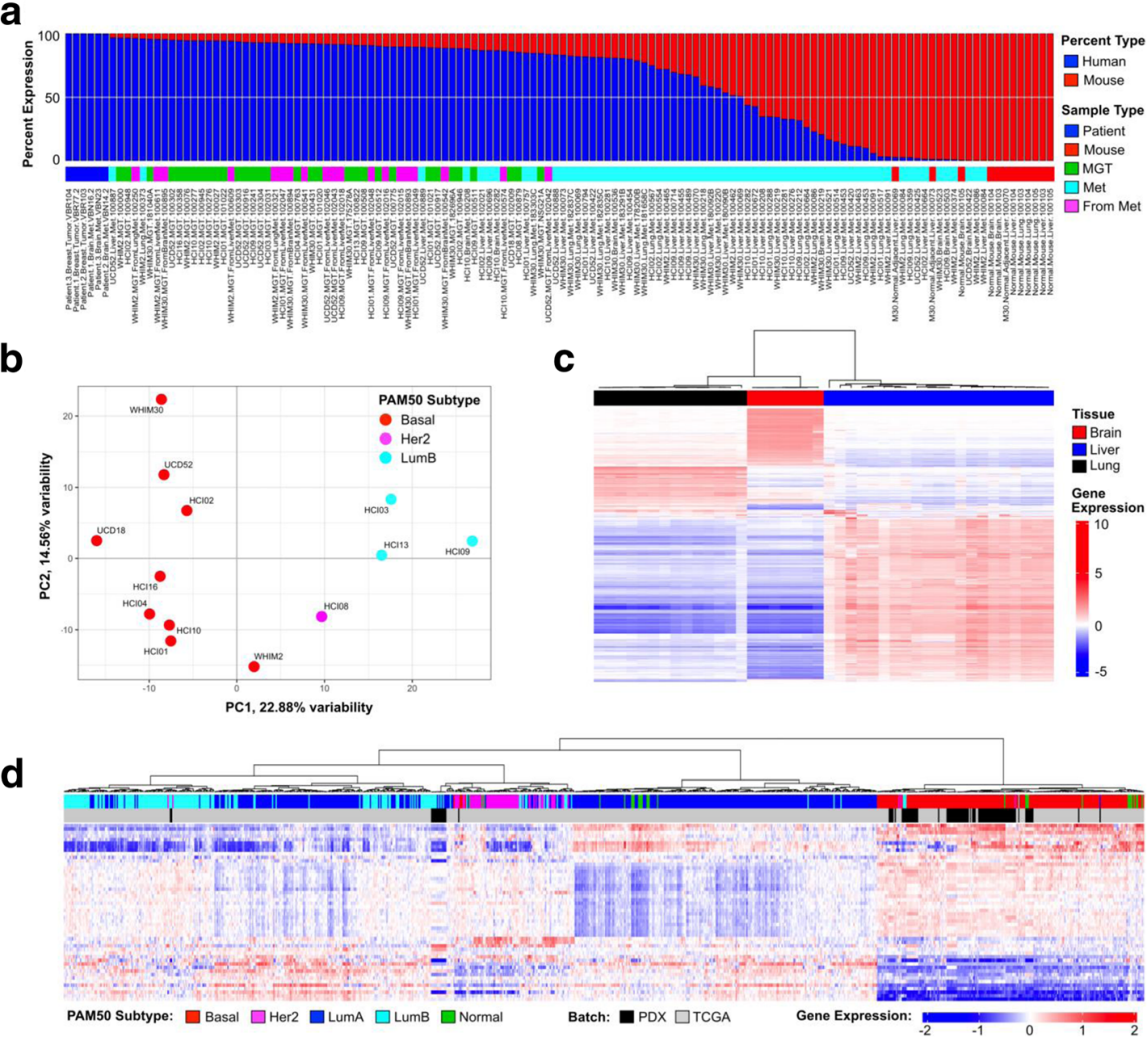
Virtual dissection of PDX mammary tumors and metastases into cancer (human) and microenvironment (mouse) gene expression datasets. The 134 sample RNA-sequencing dataset; PDX mammary tumors and metastases to the brain, lung, and liver; normal mouse brain, lung, and liver; 100% human breast tumors and brain metastases. **a** The transcripts from the 134 samples were segregated into human and mouse datasets; shown is the percent of each genome within each sample. **b** One mammary tumor from each PDX line was used in a Principal Component Analysis based on the 2000 most variable genes. **c** Mouse transcripts from metastases to the brain, lung, and liver, as well as normal mouse brain, lung, liver, were used to cluster the mouse genome based on the most variable top 2000 genes. **d** PDX tumors and metastases with more than ten million human mapped transcripts were combined with RNA-seq data from 817 tumors from The Cancer Genome Atlas. The PAM50 genes were used to hierarchical cluster the combined dataset

To determine the extent to which the cancer cell transcriptome, or organ transcriptome, changed when the cells were growing in the mammary gland, or as liver or lung metastases, RNA sequencing was performed on a set of 119 PDX mammary tumors and metastases, and normal mouse organs (3 replicates each of brain, liver, and lung). Six patient samples were also included as pure human sample controls. Given the relative lack of data on liver metastases as compared to metastases to the brain or lung, emphasis was placed on sampling disseminated cells in the liver, and only TNBC metastases were sequenced. Since the mouse and human genomes differ in their RNA sequences, we used these sequence differences to computationally separate the mouse and human genomes from each sample to generate a “mouse dataset” and a “human dataset.” Figure 3a shows the distribution of mouse and human content in the 134 samples; dataset characteristics are presented in Additional file 1. The mammary gland tumors (MGT) were 72–91% human, whereas metastases to the brain, liver, and lung  exhibited a very large range in the amount of human RNA per sample; brain (0.12–84%), liver (0.94–91%), and lung (2–90%), which is likely due to the amount of normal adjacent mouse tissue within the specimen. To determine the genomic relationships between each mammary tumor PDX, a principal component analysis was performed on the 2000 most variable genes; the tumors segregate based largely on their PAM50-defined intrinsic subtype [41]. As expected, the two ER+ PDXs that were sequenced, HCI03 and HCI13, were similar to each other and distinct from the basal-like TNBC, except for HCI09, which has been classified previously as histologically TNBC, but genetically luminal B [42] (Fig. 3b). Next, the mouse dataset was hierarchically clustered using the 2000 most variable genes from the metastases and normal mouse tissue. As expected, the lung, liver, and brain transcripts separated from each other, with some variability arising in some of the tissues harboring metastases (Fig. 3c). To determine if these models were representative of human patient samples, the human transcripts from the PDX data were merged with 817 TCGA breast cancer samples using a similar methodology as presented in Sachs et al. [43]. These data showed that the ER+ PDX integrate within the luminal TCGA samples, the HER2+ HCI08 was related to HER2-Enriched samples, and the TNBC/basal-like PDX samples had genomic profiles similar to the basal-like samples (Fig. 3d).

### Effect of microenvironment on gene expression

**Fig. 4:**
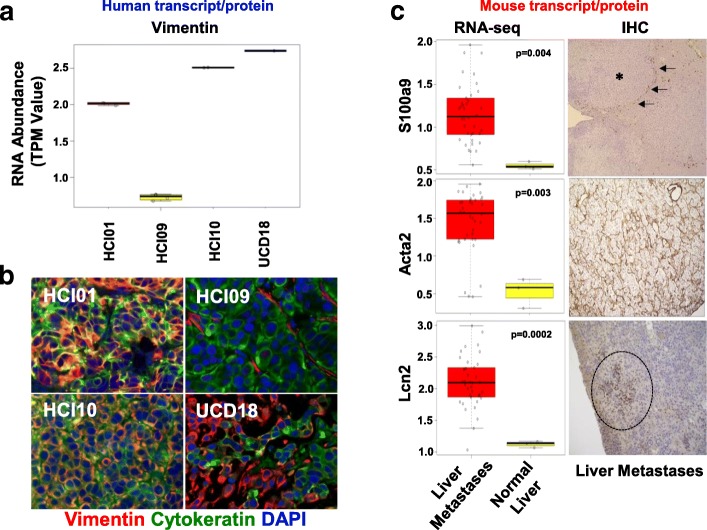
Validation of human and mouse-specific transcripts with immunofluorescence microscopy and immunohistochemistry. **a** Box and whisker plots showing human vimentin RNA expression levels in TNBC mammary tumors. **b** Immunofluorescence microscopy for pan-cytokeratin (green), vimentin (red), and DAPI (blue) in TNBC mammary tumors. **c** The mouse RNA-seq dataset was queried for genes that were differentially expressed in normal mouse liver as compared to mouse livers colonized by metastatic cells. Immunohistochemistry of liver metastases to validate the increased RNA expression observed in liver metastases. Asterisk denotes the location of cancer cells; arrows denote S100a9 cells in the peri-tumor area surrounding the cancer cells

To determine if the cancer and microenvironment datasets had been properly segregated, genes that were differentially expressed were validated by immunofluorescence or immunohistochemistry (Fig. 4). For the human dataset, vimentin expression was found to be variable across four TNBC PDX mammary tumors at the RNA level (Fig. 4a). Immunofluorescence of tumor sections for vimentin and pan-cytokeratin was congruent with the RNA-level data (Fig. 4b). HCI09 did not express vimentin other than the supporting fibroblasts, whereas UCD18 was found to have cells that expressed either cytokeratin or vimentin. HCI01 and HCI10 co-expressed both proteins in most of the tumor cells. Analysis of normal and metastatic liver samples from the mouse dataset, which represent the liver microenvironment, focused on known tissue microenvironment proteins S100a9, Acta2 (smooth muscle actin), and Lcn2 (Fig. 4c); all three of these transcripts were upregulated during metastatic growth in the liver. Immunohistochemistry for S100a9, which has been shown to be a marker for neutrophils [44], found that S100a9+ cells were often surrounding metastatic lesions in the liver, but were rarely within the bulk of the metastases. Acta2 was dramatically upregulated at the RNA and protein levels and identified fibroblasts that become activated within expanding liver metastases. Lcn2-positive cells were observed in peri-tumor areas of liver metastases.

Having confidence that the virtual dissection of cancer and microenvironment was performed properly, DESeq2 was utilized to identify genes differentially expressed in seven different TNBC PDXs when growing as a liver metastasis as compared to a mammary tumor (Additional file 6). The majority of PDXs had between 33 and 295 genes significantly upregulated and 5 to 393 significantly downregulated (*p* < 0.01) in the liver metastases as compared to the mammary tumor; in contrast, the UCD52 PDX that was serially passaged in the liver (UCD52) had more than 2000 genes significantly increased or decreased compared to the parental mammary tumor. Shown in Additional file 7a are 18 genes that were upregulated at least twofold in liver metastases compared to mammary tumors in 5 of the 7 TNBC PDXs. Ingenuity Pathway Analysis (IPA) revealed that many of these genes are known to interact and be associated with cancer (Additional file 7b). In contrast to the cancer cell genomic data, when the liver microenvironment was colonized, there was a uniform and significant genomic response by the host organ to each of the PDXs; 27 genes were significantly upregulated in the mouse liver of every PDX (Additional file 7c). IPA analyses of these 27 genes (the liver microenvironment-induced gene signature) revealed that their top biological function is as mediators of the inflammatory response (Additional file 7d). The genes upregulated in the mouse liver genome in response to PDX cells are shown in Additional file 8.

A more limited approach was taken to investigate the genes which were dynamically expressed in lung metastases. When comparing mammary tumors and lung metastases from three TNBC PDXs, similar trends were observed as with the liver analyses, with different magnitudes of gene expression being observed across the PDXs (Additional file 9 and Additional file 10). Interestingly, only two human genes were found to be upregulated in most liver metastases and lung metastases: A2M and ST3GAL5. For the mouse lung genomic response to metastatic growth, there were 3656 genes upregulated more than twofold in 2 of 3 PDX lung metastases (Additional file 11). In contrast to the few common human genomic changes in liver and lung metastases, there were 299 transcripts similarly upregulated in mouse liver and lung RNA, which were likely in part due to transitionary neutrophils and macrophages. Overall, across all the PDXs tested, the liver genomic response to the invading cancer cells was more robust and consistent than that of the lung.

### Separation of liver-expressed transcripts from human metastasis gene signatures

**Fig. 5:**
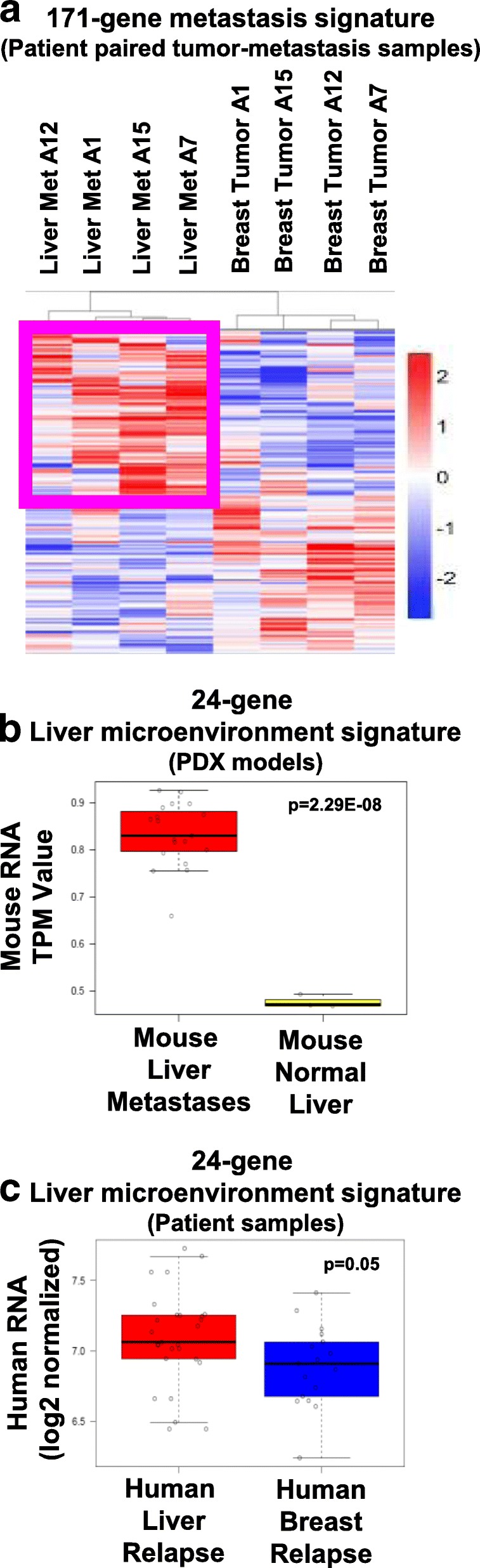
Identification of genes within human metastasis signatures that originate from liver cells. **a** RNA-seq data from patient-matched breast tumors and liver metastases were downloaded from Siegel et al. [30], median centered, and hierarchically clustered. Highlighted in the purple box are 171 genes increased in each liver metastasis compared to the primary tumor it was derived from. **b** ANOVA was performed on the PDX mouse RNA-seq dataset for all 171 genes to identify the transcripts which were induced in the liver cell transcriptome during PDX metastasis compared to normal mouse liver. Twenty-four liver genes were identified as significantly upregulated (*p* < 0.05) in metastases compared to normal liver and were averaged to generate a signature value for each sample. PDX liver macro-metastases were used for the analyses that had human RNA content > 50% (*n* = 19), normal mouse liver (*n* = 3). **c** 27 liver relapses and 17 breast relapse fine-needle aspirates from Tobin et al. [31] were queried for the 27-gene liver microenvironment gene signature presented in Additional file 7c

The next goal was to determine if this data could be used to distinguish cancer and organ-specific genes from metastasis biopsies. To identify genes that were differentially expressed in matched tumors and metastases, Siegel et al. [30] compared the DNA and RNA of primary breast tumors and their matched metastases from autopsy samples and identified a set of genes that were differentially expressed in each tumor-metastasis pair; 171 genes were increased in metastases (Fig. 5a). We tested the 171 genes to identify those that were likely arising from the liver microenvironment. Analysis of variance tests on the mouse RNA-seq dataset, comparing PDX liver metastases with normal mouse liver, revealed that 24 of the 171 genes were significantly (*p* < 0.05) upregulated in the liver transcriptome from the PDX samples (Fig. 5b), suggesting these genes might be derived from the liver microenvironment in response to cancer growth. Analysis of a second dataset of human breast cancer fine-needle aspirates (FNA) from liver or breast relapses [31] found that the activated liver microenvironment signature shown in Additional file 7c was significantly higher in the liver relapses (Fig. 5c); hence, even in FNA samples, contributions of the microenvironment genome can be identified.

### Distinguishing clonal selection of metastatic cells and microenvironment-induced temporary genomic changes in cancer cells

**Fig. 6:**
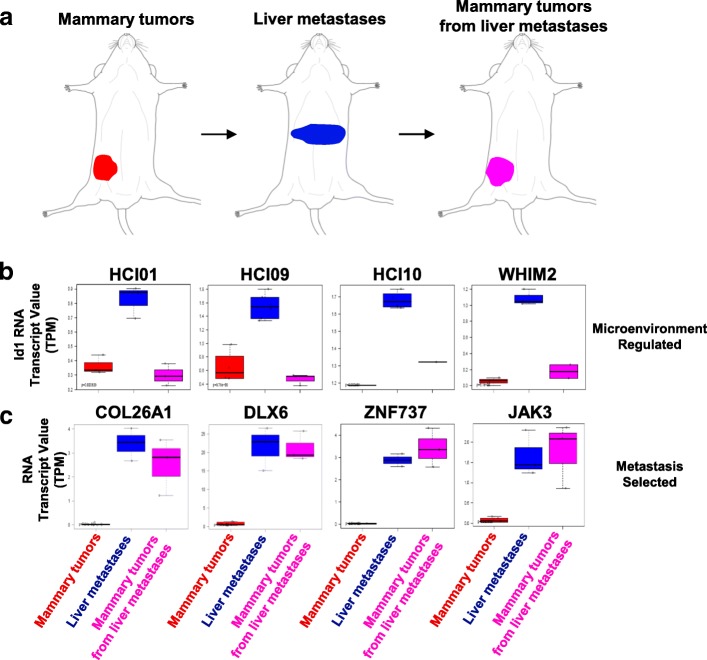
Identification of microenvironment-regulated genes or metastasis-selected genes. **a** Approach used to compare gene expression profiles of mammary gland tumors (MGT), liver metastases, and MGT which were grown from liver metastases. **b** Human ID1 gene expression levels in MGT, metastases, and MGT grown from metastases. Most metastases have increased ID1 expression, which reverts to levels observed in parental MGT when the metastases are collected and regrown in the mammary gland. **c** Shown are examples of genes upregulated in UCD52 liver metastases, which maintained high expression when the liver metastases were collected and grown in the mammary gland

To determine the pathways that were permanently altered, or selected for, during metastasis, as compared to the genes that were temporarily increased in the cancer cells due to the liver or lung microenvironment, sets of metastases were collected and transplanted back into the mammary gland (Fig. 6a). Triads of tumors (i.e., mammary tumors, metastases, and mammary tumors from metastases) were RNA-sequenced and compared. Microenvironment-induced plasticity was observed in some genes, such as Id1, that were upregulated in metastases, but then reverted to levels observed in the parental mammary tumors when liver metastases were collected and regrown in the mammary gland (Fig. 6b). When twofold change cutoff rules were applied, there were 54, 53, 123, and 19 genes within HCI01, HCI09, UCD52, and WHIM2, respectively, that fit this microenvironment-induced pattern. In contrast, within the UCD52 PDX that was selected for liver-tropism, 810 genes were found to be upregulated at least twofold in liver metastases, and they remained highly expressed when the metastatic cells were collected and regrown in the mammary gland (Fig. 6c); there were 18, 59, and 35 genes, for HCI01, HCI09, and WHIM2, respectively, that were permanently upregulated after metastasis selection. Collectively, these results provide evidence that clonal selection of cells most fit to survive in organ microenvironments occurs and that the microenvironment induces temporary genomic changes in the cancer cells.

### Genetic signatures activated in basal-like metastases

**Fig. 7:**
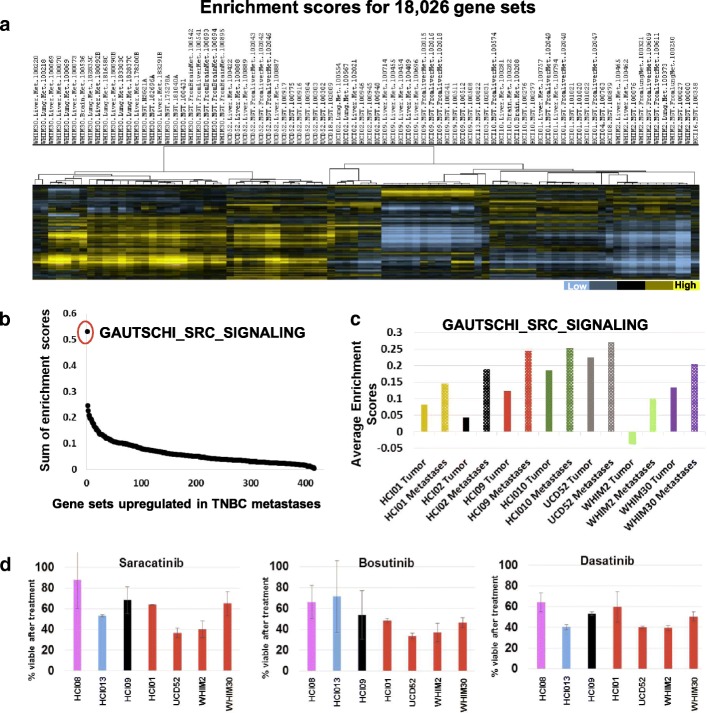
Gene Set Enrichment Analyses identify signatures upregulated in metastases. **a** Gene set enrichment scores for tumors and metastases were identified for 18,026 gene signatures and hierarchical clustered. **b** > 400 gene sets were found to be upregulated on average in metastases from all six basal-like PDXs, the sum of enrichment scores was identified by adding together the difference in enrichment scores of all 6 PDXs (metastases compared to tumors). **c** Average enrichment score values for one gene set across different tumors and metastases. **d** Effect of targeting the most upregulated pathway with three different Src inhibitors on viability of PDX spheroids in suspension culture

Since every tumor has some variability in baseline gene expression compared to other tumors, we were curious whether broader biological pathway analyses would reveal genomic programs shared by basal-like PDXs when they metastasize. Therefore, single-sample Gene Set Enrichment Analysis was performed with 18,026 gene sets from the Molecular Signatures Database. Hierarchical clustering of the enrichment scores for each sample revealed that every PDX was unique, and the metastases had a strong correlation with mammary tumors (Fig. 7a). Shown in Fig. 7b are the 416 gene sets that had higher enrichment scores in basal-like liver and lung metastases compared to mammary tumors. To identify the “sum of enrichment scores” value, the difference in enrichment scores between tumors and metastases for all six basal-like PDXs were added together. The GAUTSCHI_SRC_SIGNALING gene set [45] was found to be the most activated in metastases across all the PDXs (Fig. 7c); the top two additional pathways that shared overlapping genes with this gene set include KEGG_TGF_BETA_SIGNALING_PATHWAY and PLASARI_TGFB1_TARGETS_1HR_UP. Both these signatures had higher enrichment scores in metastases compared to mammary tumors (in 5 of 6 basal-like PDXs). To determine if targeting SRC signaling would have cytotoxic effects on the PDXs, spheroids were generated from mammary tumors and treated with 3 SRC inhibitors. Each inhibitor exhibited cytotoxic activity on the basal-like models (Fig. 7d) which provide justification for further in vivo testing in the metastatic setting. However, since SRC inhibitors have thus far failed as single agents for TNBC [46, 47], our ongoing efforts are aimed at identifying synergistic combinations with SRC inhibitors and other drugs to test on metastases.

## Discussion

We sought to use PDX models to determine how organ microenvironments change in parallel to cancer transcriptomic variations during metastasis. The ability to separate the PDX RNA-seq data into human (cancer) and mouse (microenvironment) transcriptomic datasets allowed for a clearer understanding of how each tissue compartment contributes to gene profiles associated with metastatic growth. Analysis of the cancer genomic data identified > 400 pathways that had higher enrichment scores in metastases from most or all the basal-like PDXs. In 2015, Lawson et al. utilized the HCI01, HCI02, and HCI10 PDXs and demonstrated that each PDX could generate lung metastases from a mammary tumor [48]; at the transcriptional level, the early metastatic cells possessed a distinct stem-like gene expression signature, whereas high-burden metastases became more similar to the primary tumor. We also observed many stem-cell, epithelial-to-mesenchymal transition, and TGF-beta-related signatures being some of the gene signatures most highly upregulated in the metastases. We did note that when attempting to identify genomic differences in primary tumors and micro- or macro-metastases, the analyses could be influenced by differences in the metastases themselves, fluctuating proportions of transitory cells, as well as the read depth of the sequencing. The extent or depth of sequencing per sample has been shown to be negatively correlated with the robustness of differential gene expression detection [49], and the average false discovery rate of differently expressed genes increases as read depth decreases. This implies that the higher the read depth the more robust a differently expressed gene list will be, especially for genes expressed at a relatively low level. For PAM50 subtyping, we found that at least 10 M reads were ideal for tumor classification. Some of the smaller micrometastasis samples analyzed contained low human read counts; however, due to limited numbers of samples for each PDX, for some contrasts, we chose to utilize all data for DE analysis. Moving forward, single-cell level information will become important to analyze and contrast with genomic insights from bulk tissue studies.

The genomic response of the liver microenvironment cells in response to the cancer cells was similar for the majority of the basal-like PDXs. The inflammatory response gene profile was due in part to recruited S100A9+ neutrophils which have been shown to be important for metastasis [44, 50]. It is likely that some of the biological pathways activated within the metastatic microenvironment could be non-invasively screened for in blood samples from breast cancer patients as a non-invasive assessment for initiation or growth of a breast cancer liver metastasis; examples of secreted liver factors that were upregulated in response to metastases include NPY, VGF, SCG5, and CXCL1.

Biopsies of metastases are now being utilized to guide therapeutic selection. The data we present in this study revealed that 14% of a metastasis signature obtained from human liver metastases were genes that were activated in the liver microenvironment in response to cancer invasion. A second human metastasis genetic dataset also confirmed the influence of the microenvironment on cancer genomic profile. Therefore, as we seek to develop therapeutics that target both the cancer cells and the microenvironment, these data, and more broadly these approaches, can be used to determine the pathways in both tissue compartments that mediate viability. Src signaling was identified as highly activated in metastases; however, clinically, these inhibitors have failed to prove effective as monotherapies and often lead to tumor reprogramming to continue growing. Therefore, we hypothesize that the best anti-metastasis therapies will be synergistic drug combinations that target SRC in addition to other metastasis-promoting programs identified in these studies.

## Conclusions

In this set of studies, we sought to increase our understanding of the genomic programs that contribute to successful breast cancer metastasis. Since the growth of metastatic cells depends on the characteristics of the cancer cells, as well as the response of the organ being colonized, we aimed to elucidate parallel mechanisms from both tissue compartments. Through various experimental and computational approaches using human tumors within immunocompromised mice, the lung was found to be the most common site of relapse; lymph nodes and liver were the other most common metastatic sites. The majority of a PDX genomic profile was maintained when the PDX was growing in the liver, lung, or brain, yet common genetic programs were identified that were activated in the metastatic setting; targeting of this pathway with Src inhibitors resulted in cytotoxicity to the basal-like PDX spheroids. Multiple pathways within the cancer cells, and potentially within the host organ as well, may need to be targeted to inhibit the growth of metastases. The consistent response by the microenvironment highlights that this tumor compartment warrants more attention for anti-metastasis therapy.

## Additional files

Additional file 1: RNA-seq dataset characteristics. (XLSX 38 kb)

Additional file 2: Assessment of estrogen receptor (ER), progesterone receptor (PR), and HER2 expression in 14 patient-derived xenograft models used in this study. Three mammary tumors from each patient-derived xenograft were extracted at similar size, paraffin embedded, and sectioned. Representative 40x images from each line are shown. TNBC; HCI01, HCI02, HCI09, HCI10, HCI16, UCD18, UCD52, WHIM2, WHIM30. ER+/PR+; HCI03, HCI11, HCI13. HER2+; HCI04 (weak), HCI08. The PR+ cells in WHIM2 and WHIM30 are due to cross reactivity of the antibody and mouse mammary epithelial duct. (PDF 449 kb)

Additional file 3: Characterization of metastatic properties of Patient Derived Xenograft models after intracardiac injection. **(a)** 500,000 WHIM2 + GFPLuc or WHIM30 + GFPLuc cells were injected into the left ventricle of NSG mice and mice were monitored weekly for appearance of metastases. **(b)** Overall survival plot for mice injected with WHIM2 (cohort 1; *n* = 6, cohort 2; *n* = 13) or WHIM30 (cohort 1; *n* = 9, cohort 2; *n* = 9) PDX cells. (PDF 140 kb)

Additional file 4: Comparison of organs with metastatic cells from the reported patient data and these studies. (PDF 174 kb)

Additional file 5: Overview of approach used to generate UCD52 liver tropic metastases. (PDF 86 kb)

Additional file 6: Excel file with DESeq2 results comparing mammary tumors with liver metastases. (XLSX 28591 kb)

Additional file 7: Genes upregulated during liver metastasis; cancer and organ-specific changes. DESeq2 was used to identify RNA transcripts that were **(a)** upregulated in human genes (> 1.5 fold in 5 of 7 PDX) in liver metastases compared to mammary gland tumors. **(b)** the top scoring Ingenuity Pathway Analysis network from the human dataset. **(c)** RNA transcripts upregulated in mouse genes (> 2 fold in 6 of 7 PDX) in liver metastases compared to normal liver in the mouse RNA-seq dataset. In the human dataset asterisks denote transcripts significantly different false discover rate (FDR) < 0.05. All genes displayed in the mouse dataset are FDR < 0.05. **(d)** the top scoring Ingenuity Pathway Analysis network from the mouse dataset. (PDF 402 kb)

Additional file 8: Excel file of DESeq2 results of the mouse RNA dataset comparing normal liver vs liver metastases. (XLSX 4356 kb)

Additional file 9: Genes upregulated during lung metastasis; cancer and organ-specific changes. DESeq2 was used to identify RNA transcripts that were upregulated in human genes (> 1.5 fold in 2 of 3 PDX) in lung metastases compared to mammary gland tumors or upregulated in mouse genes (> 2 fold in 3 of 3 PDX) in lung metastases compared to normal lung in the mouse RNA-seq dataset. Shown are the top 50 genes upregulated on average for each comparison. (PDF 77 kb)

Additional file 10: Excel file of DESeq2 results of mammary tumors compared to lung metastases. (XLSX 7698 kb)

Additional file 11: Excel file of DESeq2 results of the mouse RNA dataset comparing normal lung vs lung metastases. (XLSX 2299 kb)

## Abbreviations

ER: Estrogen receptor
FNA: Fine-needle aspirate
GFP: Green fluorescent protein
GSEA: Gene Set Enrichment Analyses
HCI: Huntsman Cancer Institute
HER2: Human epidermal growth factor receptor 2
IPA: Ingenuity Pathway Analysis
Luc: Luciferase
PCA: Principal component analysis
PDX: Patient-derived xenograft
PR: Progesterone receptor
TNBC: Triple-negative breast cancer
UCD: University of Colorado Denver
WHIM: Washington University Human in Mouse
