# Supplementary figures and images for: Separation of breast cancer and organ microenvironment transcriptomes in metastases
Source: Breast Cancer Res. 2019 Mar 6;21:36. doi: 10.1186/s13058-019-1123-2 (PMC6404325; doi:10.1186/s13058-019-1123-2)

Additional File 2

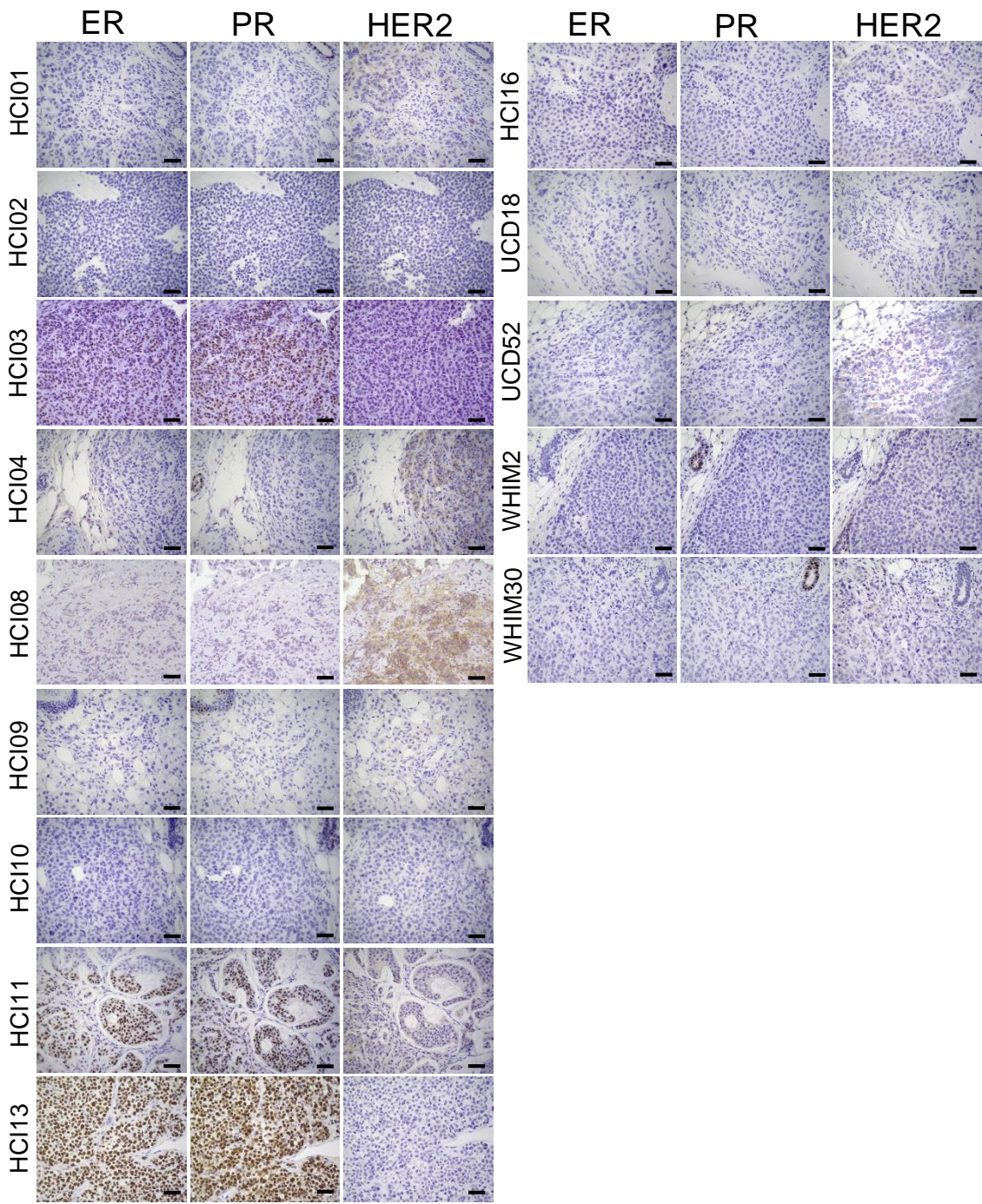

Supplement: Supplementary file 2 — Assessment of estrogen receptor (ER), progesterone receptor (PR), and HER2 expression in 14 patient-derived xenograft models used in this study. Three mammary tumors from each patient-derived xenograft were extracted at similar size, paraffin embedded, and sectioned. Representative 40x images from each line are shown. TNBC; HCI01, HCI02, HCI09, HCI10, HCI16, UCD18, UCD52, WHIM2, WHIM30. ER+/PR+; HCI03, HCI11, HCI13. HER2+; HCI04 (weak), HCI08. The PR+ cells in WHIM2 and WHIM30 are due to cross reactivity of the antibody and mouse mammary epithelial duct. (PDF 449 kb) [file 13058_2019_1123_MOESM2_ESM.pdf]

a

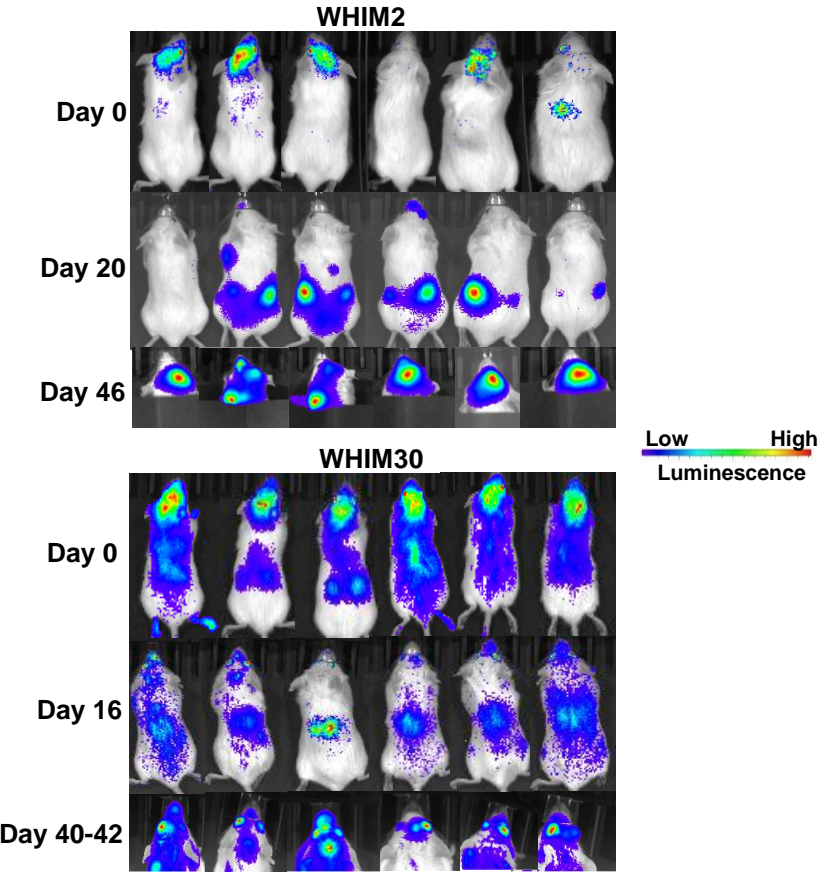

b

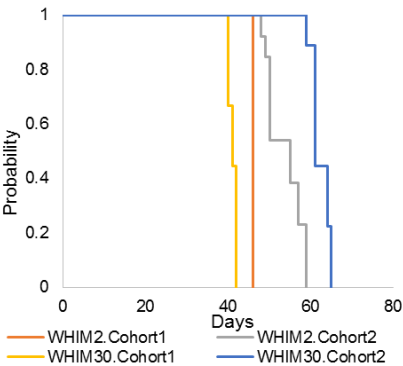

Supplement: Supplementary file 3 — Characterization of metastatic properties of Patient Derived Xenograft models after intracardiac injection. (a) 500,000 WHIM2 + GFPLuc or WHIM30 + GFPLuc cells were injected into the left ventricle of NSG mice and mice were monitored weekly for appearance of metastases. (b) Overall survival plot for mice injected with WHIM2 (cohort 1; n = 6, cohort 2; n = 13) or WHIM30 (cohort 1; n = 9, cohort 2; n = 9) PDX cells. (PDF 140 kb) [file 13058_2019_1123_MOESM3_ESM.pdf]

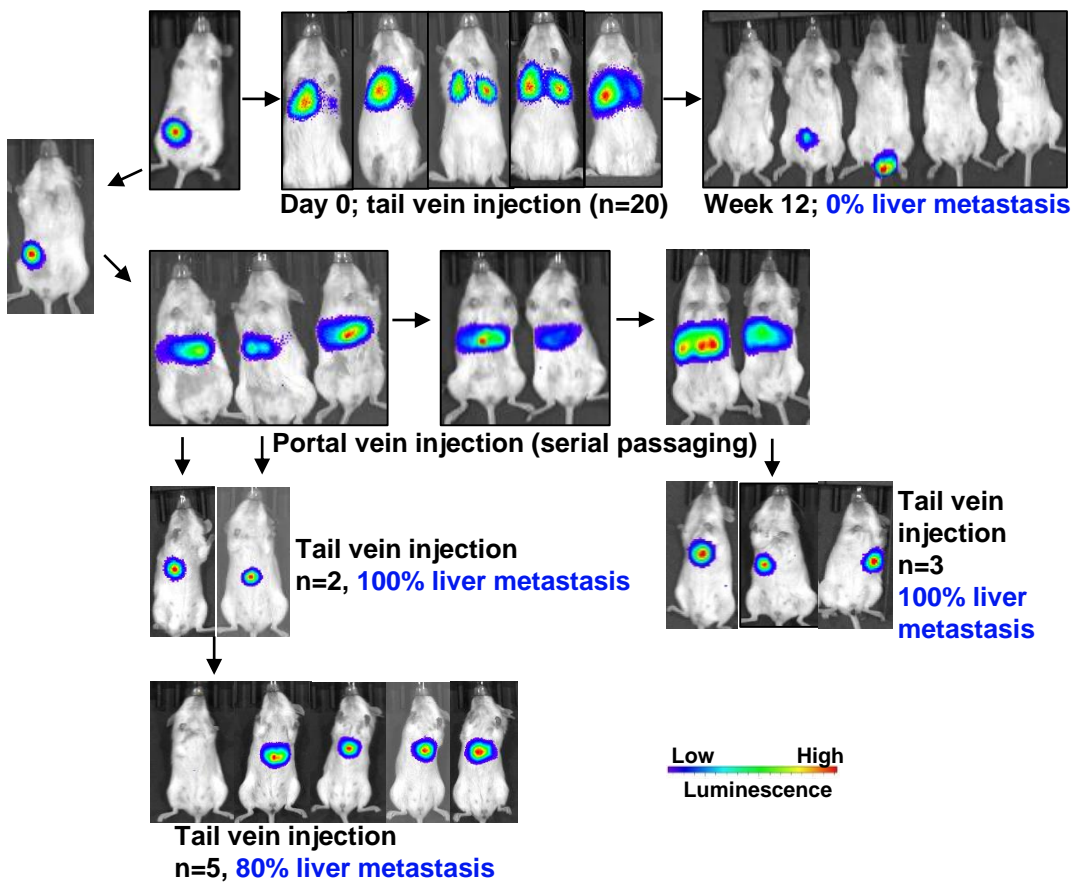

Supplement: Supplementary file 5 — Overview of approach used to generate UCD52 liver tropic metastases. (PDF 86 kb) [file 13058_2019_1123_MOESM5_ESM.pdf]
