# Supplementary material for: Separation of breast cancer and organ microenvironment transcriptomes in metastases
Source: Breast Cancer Res. 2019 Mar 6;21:36. doi: 10.1186/s13058-019-1123-2 (PMC6404325; doi:10.1186/s13058-019-1123-2)
Supplement: Supplementary file 4 — Comparison of organs with metastatic cells from the reported patient data and these studies. (PDF 174 kb) [file 13058_2019_1123_MOESM4_ESM.pdf]

Additional File 4

PDX	Organs of relapse		
	Patient metastases	PDX-metastases from mammary tumor	PDX-metastases after tail-vein
HCI01	lung	liver, lung, lymph node	liver, ovary
HCI02	lymph node	lung, lymph node	liver, lung
HCI03	lymph node	liver, lung, lymph node	not determined
HCI04	not detected	not detected	not detected
HCI08	lung, skin	lung	lung
HCI09	bone, lymph node, pancreas, peritoneum	liver, lung, lymph node	liver, lung, ovary
HCI10	lung	brain, liver, lymph node, lung, ovary	brain, liver, lung, ovary
HCI11	lymph node, pleura	lung	brain, liver, lymph node, lung
HCI13	liver, lung, pericardium	brain, lung	lung, lymph node
HCI16	unknown	not detected	not detected
UCD18	unknown	not detected	not detected
UCD52	unknown	lymph node, lung	uterus
WHIM2	brain, bone, lymph nodes	brain, liver, lymph nodes, ovary, salivary gland	brain, liver, lung, lymph node, ovary, salivary gland
WHIM30	not detected	brain, liver, lung	brain, liver, lung
