# Supplementary material for: Separation of breast cancer and organ microenvironment transcriptomes in metastases
Source: Breast Cancer Res. 2019 Mar 6;21:36. doi: 10.1186/s13058-019-1123-2 (PMC6404325; doi:10.1186/s13058-019-1123-2)

Additional File 7

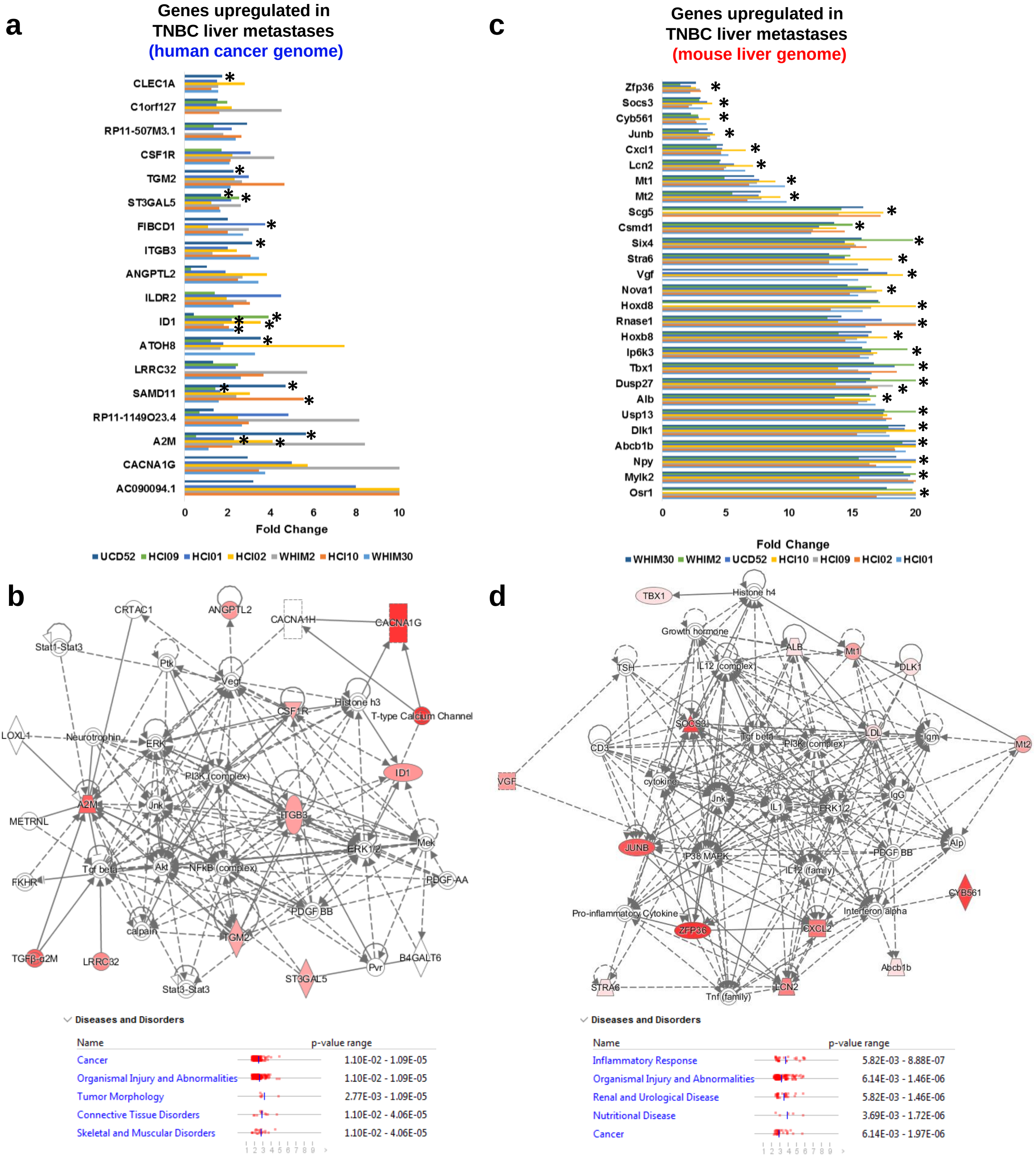

Supplement: Supplementary file 7 — Genes upregulated during liver metastasis; cancer and organ-specific changes. DESeq2 was used to identify RNA transcripts that were (a) upregulated in human genes (> 1.5 fold in 5 of 7 PDX) in liver metastases compared to mammary gland tumors. (b) the top scoring Ingenuity Pathway Analysis network from the human dataset. (c) RNA transcripts upregulated in mouse genes (> 2 fold in 6 of 7 PDX) in liver metastases compared to normal liver in the mouse RNA-seq dataset. In the human dataset asterisks denote transcripts significantly different false discover rate (FDR) < 0.05. All genes displayed in the mouse dataset are FDR < 0.05. (d) the top scoring Ingenuity Pathway Analysis network from the mouse dataset. (PDF 402 kb) [file 13058_2019_1123_MOESM7_ESM.pdf]
