# Supplementary material for: Separation of breast cancer and organ microenvironment transcriptomes in metastases
Source: Breast Cancer Res. 2019 Mar 6;21:36. doi: 10.1186/s13058-019-1123-2 (PMC6404325; doi:10.1186/s13058-019-1123-2)

Additional File 9

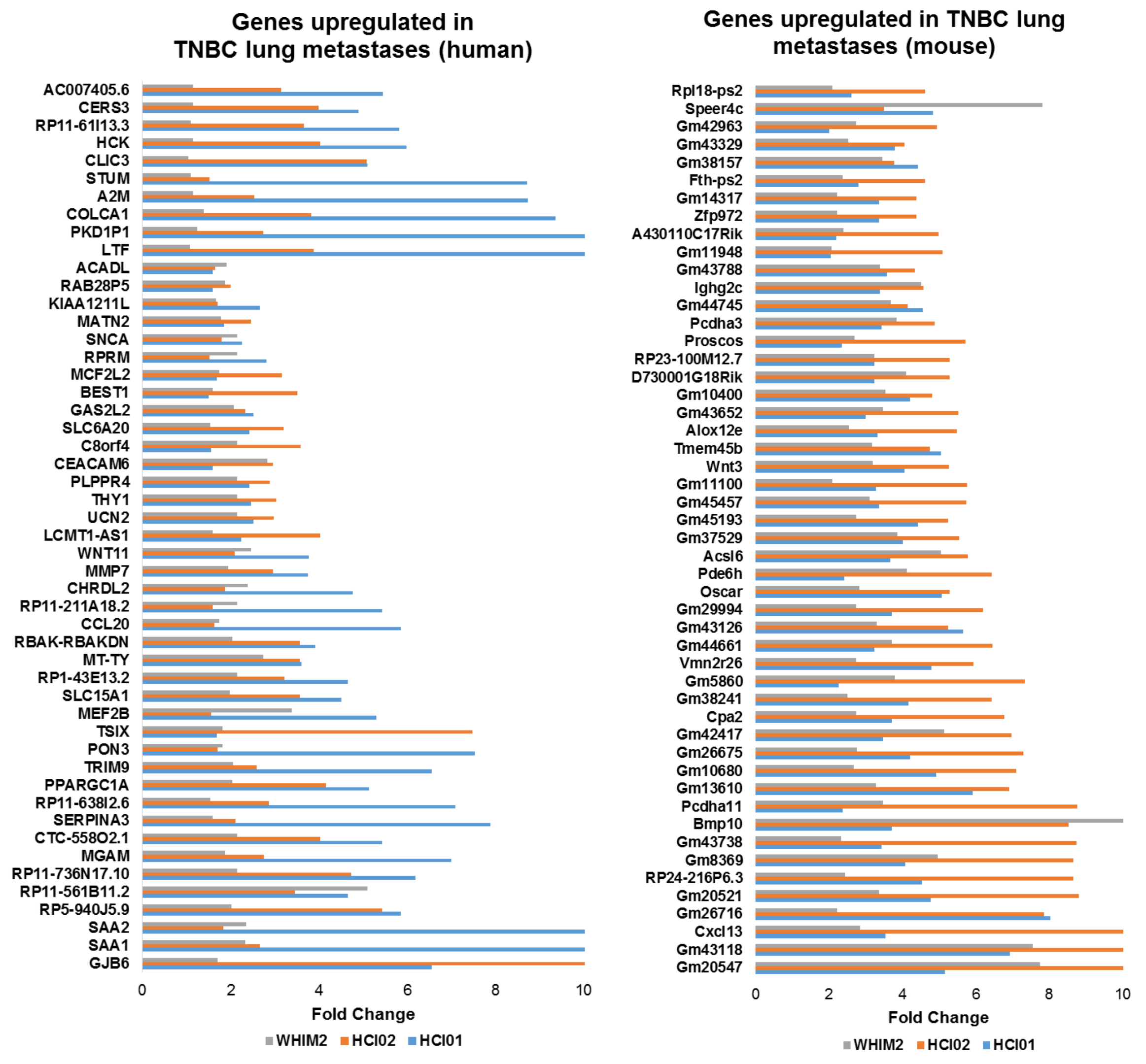

Supplement: Supplementary file 9 — Genes upregulated during lung metastasis; cancer and organ-specific changes. DESeq2 was used to identify RNA transcripts that were upregulated in human genes (> 1.5 fold in 2 of 3 PDX) in lung metastases compared to mammary gland tumors or upregulated in mouse genes (> 2 fold in 3 of 3 PDX) in lung metastases compared to normal lung in the mouse RNA-seq dataset. Shown are the top 50 genes upregulated on average for each comparison. (PDF 77 kb) [file 13058_2019_1123_MOESM9_ESM.pdf]
